# Cluster randomized trial of a mHealth intervention “ImTeCHO” to improve delivery of proven maternal, neonatal, and child care interventions through community-based Accredited Social Health Activists (ASHAs) by enhancing their motivation and strengthening supervision in tribal areas of Gujarat, India: study protocol for a randomized controlled trial

**DOI:** 10.1186/s13063-017-1998-0

**Published:** 2017-06-09

**Authors:** Dhiren Modi, Shrey Desai, Kapilkumar Dave, Shobha Shah, Gayatri Desai, Nishith Dholakia, Ravi Gopalan, Pankaj Shah

**Affiliations:** 1 Community Health Department, SEWA-Rural, Jhagadia, District, Bharuch, Gujarat 393110 India; 2 Commissionerate of Health, Government of Gujarat, Block 5, Dr. Jivraj Mehta Bhavan, Gandhinagar, Gujarat 382010 India; 3 Argusoft India Ltd., GIDC electronic estate, Sector 25, Gandhinagar, 382016 Gujarat India

**Keywords:** mHealth, ASHAs, cRCT, Maternal health, Neonatal health, Acceptability, Tribal health, Implementation research, India

## Abstract

**Background:**

To facilitate the delivery of proven maternal, neonatal, and child health (MNCH) services, a new cadre of village-based frontline workers, called the Accredited Social Health Activists (ASHAs), was created in 2005 under the aegis of the National Rural Health Mission in India. Evaluations have noted that coverage of selected MNCH services to be delivered by the ASHAs is low. Reasons for low coverage are inadequate supervision and support to ASHAs apart from insufficient skills, poor quality of training, and complexity of tasks to be performed. The proposed study aims to implement and evaluate an innovative intervention based on mobile phone technology (mHealth) to improve the performance of ASHAs through better supervision and support in predominantly tribal and rural communities of Gujarat, India.

**Methods/design:**

This is a two-arm, stratified, cluster randomized trial of 36 months in which the units of randomization will be Primary Health Centers (PHCs). There are 11 PHCs in each arm. The intervention is a newly built mobile phone application used in the public health system and evaluated in three ways: (1) mobile phone as a job aid to ASHAs to increase coverage of MNCH services; (2) mobile phone as a job aid to ASHAs and Auxiliary Nurse Midwives (ANMs) to increase coverage of care among complicated cases by facilitating referrals, if indicated and home-based care; (3) web interface as a job aid for medical officers and PHC staff to improve supervision and support to the ASHA program. Participants of the study are pregnant women, mothers, infants, ASHAs, and PHC staff.

Primary outcome measures are a composite index made of critical, proven MNCH services and the proportion of neonates who were visited by ASHAs at home within the first week of birth. Secondary outcomes include coverage of selected MNCH services and care sought by complicated cases. Outcomes will be measured by conducting household surveys at baseline and post-intervention which will be compared with usual practice in the control area, where the current level of services provided by the government will continue. The primary analysis will be intention to treat.

**Discussion:**

This study will help answer some critical questions about the effectiveness and feasibility of implementing an mHealth solution in an area of MNCH services.

**Trial registration:**

Clinical Trial Registry of India, CTRI/2015/06/005847. Registered on 3 June 2015.

**Electronic supplementary material:**

The online version of this article (doi:10.1186/s13063-017-1998-0) contains supplementary material, which is available to authorized users.

## Background

India carries the largest burden of maternal, neonatal and infant mortality, and malnutrition in the world [1–3]. Many of the deaths can be prevented with the help of proven, cost-effective, community-based services [4, 5]. Some of the proven maternal, neonatal, and child health (MNCH) services include antenatal examination, supplementation of iron and folic acid during pregnancy, promotion of skilled birth attendance, home-based neonatal care, and early and exclusive breast feeding during the first 6 months, initiation of complementary feeding at the completion of 6 months, supplementation of vitamin A among infants, case management of pneumonia, and oral rehydration therapy for diarrhea [4–6].

To increase the coverage of proven MNCH services, a new cadre of village-based frontline workers, called the Accredited Social Health Activists (ASHAs), was created by the Government of India in 2005 [7]. An ASHA is a native of a particular village and is selected by the village council with help of local government health staff. ASHAs are expected to be literate and to have an educational qualification of at least 8^th^ grade. After training, an ASHA is expected to contribute 3–5 hours every day for her activities. There is one ASHA for approximately every 1000 members of a rural population, and there were 863,503 ASHAs in India in 2013 [8]. ASHAs are supported and supervised by the staff of Primary Health Centers (PHCs). A PHC is an operational and administrative unit which provides curative and public health services for all of its designated villages. Each PHC has a team of two medical officers (doctors) and four to six Auxiliary Nurse Midwives (ANMs) along with one Lady Health Visitor and several male multi-purpose workers. ASHAs receive a performance-based incentive [9].

Unfortunately, coverage of some of the most impactful MNCH services to be delivered by ASHAs has remained low, especially in hard-to-reach tribal areas [10–13]. Some important reasons for the suboptimal performance of the ASHA program are common implementation bottlenecks such as inadequate training leading to suboptimal skills and knowledge, along with a lack of institutional capacity leading to insufficient supervision, support, and motivation of ASHAs [10, 11, 14]. To overcome these problems, an mHealth-based intervention, called "ImTeCHO," was created. ImTeCHO is a mobile phone application which aims to increase the performance of the ASHAs and PHC staff by creating a platform to improve supervision, support, and motivation. Detailed information about the development and components of the ImTeCHO intervention is described in another publication [15].

The aim of this protocol is to test the effectiveness of the ImTeCHO intervention in improving coverage of proven MNCH services through a cluster randomized trial in tribal areas of Gujarat, India. The primary objective of the trial is to examine the effect of ImTeCHO in the form of job aid to ASHAs during their scheduled home visits to increase coverage of selected MNCH services provided by the ASHAs in tribal and rural areas of Gujarat, India compared to a control area where usual care will be continued. The secondary objective is to examine the effect of ImTeCHO intervention to increase coverage of care among complicated maternal, neonatal, and child cases by facilitating referrals to health facilities and managing at home for those cases that cannot be referred. Another secondary objective is to examine the effect of ImTeCHO intervention in the form of job aid to medical officers and PHC staff to improve the support and supervision of ASHAs.

## Methods/design

### Study design

This is a two-arm, parallel, stratified cluster randomized trial in which the unit of randomization is the PHC. Because this is a complex community-based intervention, a cluster randomization is required. The ImTeCHO web interface will be primarily used by the PHC staff, including the medical officer, and other support staff; thus, using the PHC as the unit of randomization will reduce contamination between clusters. A PHC is an operational and administrative unit for providing curative and public health services for all its covered villages. Each PHC has a team of two medical officers, four to six ANMs, one Lady Health Visitor, and several male multi-purpose workers. Of the two medical officers, one has an MBBS degree and is in charge of the PHC, including clinical services. The other medical officer is usually an Ayurveda, Yoga and Naturopathy, Unani, Siddha, and Homoeopathy (AYUSH) practitioner and is involved in field-based duties. Each ANM is in charge of one subcenter which serves a population of 3000–4000. On an average, there are 20–25 ASHAs per PHC. There is one ASHA Facilitator for approximately every 10 ASHAs. The average size of the population per PHC is approximately 20,000 in tribal areas [16].

### Study setting

This study will be conducted by SEWA Rural, which is a voluntary organization located in the Jhagadia block of Gujarat. SEWA Rural has been involved in health and development activities in rural, tribal areas of Gujarat, India since 1980 [17]. The study area belongs to the tribal belt which stretches along the Satpura mountain range located along the eastern part of Gujarat. Some of the study area is covered by thick forest. The tribal population residing in the study area is called “Vasava” or “Bhil.” As with other tribal communities, the Vasava tribe has its own language and customs which are different from the mainstream culture, though this is changing rapidly as they become increasingly connected with today’s more modern culture. Agriculture is the main occupation, with a large number of the population working as landless laborers [18]. The rate of industrialization is much slower in these areas. Dediyapada, Nandod, and Sagbara belong to the Narmada district which was named one of the *most backward districts* by the Ministry of Panchayati Raj in 2006 due to its lack of development and difficult terrain [19]. The literacy rate of the study area was 65%, and the proportion of scheduled tribe population was 75% in a 2011 census of India.

### Study participants

#### ASHA

A detailed description of the ASHA is provided in the Background. Most of the ASHAs belong to the scheduled tribe community.

#### ASHA Facilitator (AF)

The AF is responsible for monitoring, facilitating, and providing supportive supervision of the ASHAs. The AF performs these duties during her regular field visits. There is one AF for every 10–20 ASHAs. The AF is the link between the ASHAs and the block-level support structure [20].

#### Auxiliary Nurse Midwife (ANM)

The ANM is a qualified health provider of vital primary health care services including those related to MNCH. In regard to the ASHA program, the ANM has the following role: she makes home visits and provides care to those complicated cases for persons who are unable to go to a health facility. Such care will include confirming the diagnosis, encouraging referral, counseling, and administering drugs, including oral antibiotics. The ANM conducts a monthly Village Health and Nutrition Day (VHND) in each of her coverage villages during which she provides important MNCH services including vaccination, antenatal examination, and growth monitoring. Along with the AF, the ANM also provides supportive supervision and support to the ASHAs. The ANM verifies the ASHA’s performance report which is used for calculating the ASHA’s performance-based incentive [16, 21].

#### Medical officer

The medical officer is overall in charge of a PHC. The medical officer’s main responsibilities are to provide curative service, manage promotive and preventive programs, give training, monitor field-level health workers, and oversee administrative tasks [16].

#### mHealth facilitator (mHF)

The mHF is an employee from SEWA Rural who facilitates and assists the people described above in using ImTeCHO. There is one mHF for every two PHCs; the mHF is stationed at a block health office for streamlining coordination with the government's health team [15].

The mHF's job responsibilities are as follows:

- Be the first contact person in case of any technology-related problems encountered by any users of ImTeCHO
- Use the Facilitator dashboard available through the ImTeCHO web interface for monitoring adherence to the ImTeCHO intervention through the use of innovative process indicators and undertaking housekeeping functions for dealing with migration of mothers, duplication in registering a case, and verification of death reported by ASHAs
- Attend monthly PHC meetings
- Make an occasional field visit to provide ongoing training/supervision/motivation to those ASHAs whose performance parameters within ImTeCHO are consistently poor
- Send a Short Message Service (SMS) text to the ANMs and medical officer to inform them about high-risk cases diagnosed by ASHAs

#### PHC support staff

Along with the medical officers, ANMs, and AF, other members of the PHC staff play supporting roles in the ASHA program. These staff members are data-entry operators, a pharmacist, supervisors, and multi-purpose male health workers. The PHC support staff are involved in calculation of the ASHA’s performance-based incentives and replenishment of supplies [16].

#### Helpline to provide telephone care

A helpline has been established at SEWA Rural. An experienced counselor provides telephone care which aims to support ASHAs and families to manage complicated cases over the phone, usually after receiving alerts from the ASHAs. The support involves the confirmation of a diagnosis made by the ASHA, promotion of household-level practices to care for complications, advising the ASHAs for optimal management of complications, counseling of families to seek care at a higher referral facility if required, and alerting the medical officer in case of emergencies [15].

#### Pregnant women and mothers of infants

Pregnant women and mothers of infants will be the direct beneficiaries of the intervention. They will be respondents for the baseline and endline surveys.

### Intervention

Detailed information about the intervention is described in another publication [15]. Table 1 shows the service delivery by study arm. This is followed by a brief description of the main component of the intervention which is use of the ImTeCHO mobile and web-based application by the ASHAs, medical officers, and PHC staff.

**Table 1:** Service delivery by study arm

	Intervention	Control
Three-day refresher training of ASHAs for maternal, neonatal, and child care (as per ASHA modules 6 and 7)	Yes	Yes
Four-day training and subsequent mentoring of ASHAs and PHC staff for use of ImTeCHO mobile phone and web application respectively	Yes	No
Use of ImTeCHO mobile and web-based application by ASHAs and PHC staff	Yes	No
Telephone care by a counselor	Yes	No
Ongoing technology support and facilitation through mHealth facilitators	Yes	No
Routine implementation of ASHA program	Yes	Yes
Modest, additional incentive to ASHAs for using ImTeCHO application linked to performance (ranging from US $6 to $13 monthly per ASHA)	Yes	No

**Fig. 1:**
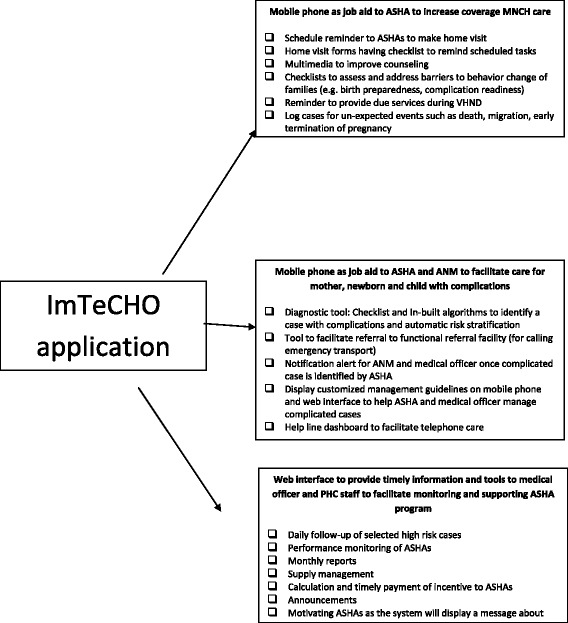
Components of the ImTeCHO mobile and web-based application

Every ASHA is given a low-cost smartphone which will be General Packet Radio Service (GPRS)-enabled, with a multi-media feature. The mobile phone application will have the following features: home visit forms, log of case details, work log, announcements, and SMS information channel. The ASHA fills out forms on her mobile during home visits which will be sent to a server using the GPRS network. Similarly, the ANMs will be given a tablet to track high-risk cases and monitor the performance of the ASHAs. Each medical officer views a web interface which is a computer screen where organized data is available online. The ImTeCHO software organizes the data into useful information (based on predefined logic) for the PHC staff. The web interface will have features to track high-risk cases, see reports, and manage incentives and supplies. No other concomitant care and interventions, apart from that mentioned above, will be delivered by the trial team; however, other organizations cannot be prohibited from introducing new public health programs, considering the nature of the trial and implementation research. See Fig. 1 for a diagram of the three main components of the mobile and web-based application.

### Outcome measures

#### Primary outcomes of interest

There are two primary outcomes of interest as follows:

1. Proportion of neonates/mothers who were visited at home by ASHA at least twice within the first week of delivery: As most of the neonatal deaths occur during the first few days of life, it is recommended that neonates should be visited three times during the first week including the day of delivery, the third day, and preferably the seventh day [22]. However, most (78% according to a coverage evaluation survey of UNICEF in 2009) of the deliveries in Gujarat now occur in a facility where the ASHA’s role is limited in the presence of the facility-based, more qualified health workers [20]. Also, the ASHA’s visit on the day of delivery is influenced by various factors with a limited role of the ImTeCHO intervention. Hence, we decided to focus on coverage of the ASHA’s two home visits within the first week of delivery after the mother and neonate return home after discharge from the facility.
2. Modified ASHA-centric composite coverage index (MACCI): The MACCI will be calculated using the following formula and rationale.
  Modified ASHA-centric composite coverage index (MACCI) = 0 · 25 × (0.33 × [Complete ASHA home visits during antenatal period + Full ANCS + FD] + [Complete HBNC] + 0 · 5 × [DPT3 + EBF] + 0 · 33 × [Care seeking for neonatal complications + ORT + ARI/febrile illness])

##### Explanation of MACCI services

1. *Maternal care services*
  Complete ASHA home visit during antenatal period = proportion of mothers who were visited at home by ASHAs at least three times during last pregnancy
  Full antenatal care service (ANCS) = proportion of mothers with full antenatal examination (at least three antenatal examinations by qualified health personnel, one tetanus toxoid injection (Inj.TT), and consumption of 100 iron-folic acid (IFA) tablets) [12]
  Facility delivery (FD) = proportion of mothers who delivered in a facility as most of the deliveries attended by skilled attendant are those taking place at a facility in Gujarat
2. *Neonatal care services*
  Complete HBNC = proportion of neonates who received the recommended number (five) of postnatal visits and at recommended times within first month of delivery by an ASHA [23]
3. *Young infant care services*
  DPT3 = proportion of infants (6–8 months old) who received three doses of diphtheria, pertussis, and tetanus vaccine or three doses of pentavalent vaccine
  EBF = proportion of infants (6–8 months old) who were exclusively breast fed for first 6 months
4. *Care seeking during complications*
  Care seeking for neonatal complications = proportion of neonates who had complications within first month of last delivery and sought care from the ASHA
  ORT = proportion of infants (6–8 months) who had diarrhea within last 2 weeks and received oral rehydration salts (ORS) from the ASHA
  ARI/fever = proportion of infants (6–8 months) with acute respiratory infection (ARI)/fever within last 2 weeks where family sought care from the ASHA

The same weight was given to each of the four main domains of interventions throughout the continuum of care: maternal, neonatal, young infant care, and care seeking for complications.

A composite outcome was required to reflect the effectiveness of ImTeCHO because the scope of the intervention is throughout the continuum of care including maternal, neonatal, and young infants up to 2 years of age. We were guided by the composite coverage index (CCI), which is now widely used to measure the coverage of key MNCH interventions and strength of the health system [24–26]. The formula for calculating the CCI was modified to develop a modified ASHA-centric composite coverage index (MACCI) for this study to focus on (1) the coverage of MNCH interventions to be provided by the ASHAs in India, (2) the scope of the ImTeCHO intervention, and (3) the relevance in India. Subsequently, ASHA visitations during antenatal and postnatal periods, exclusive breast feeding, and care seeking to the ASHAs for maternal, neonatal, and childhood complications were included, and family planning need satisfaction was omitted from the CCI formula.

#### Secondary outcomes of interest

Most of the secondary outcomes of interest are coverage of proven MNCH services to be delivered or facilitated by the ASHAs throughout the continuum of care and process indicators to assess adherence to the ImTeCHO intervention. See Table 2 for the list of primary and secondary outcomes.

**Table 2:** Primary and secondary outcomes of interest

1. Primary outcomes of interest
1.1 Proportion of neonates/mothers who were visited at home by ASHA at least twice within first week of delivery
1.2 Modified ASHA-centric Composite Coverage Index (MACCI)
2. Secondary outcomes of interest
Maternal outcomes. Proportion of:
2.1 Mothers who had first antenatal examination within first trimester
2.2 Mothers who had four or more ANC examinations by ANM/doctor including at least one examination in last trimester
2.3 Mothers with full antenatal checkup (at least three antenatal examinations, one Inj.TT, and 100 IFA tablets)
2.4 Mothers who were visited at home by ASHA at least three times during last pregnancy including at least one visit during last trimester
2.5 Mothers who had complications during last pregnancy and sought care from ASHA
2.6 Mothers who had complications within first month of last delivery and sought care from ASHA
2.7 Mothers who had serious complications during last pregnancy or within 6 weeks of last delivery and sought care from qualified health personnel
2.8 Mothers who delivered in a facility
Neonatal outcomes. Proportion of:
2.9 Mothers who initiated breast feeding within an hour of last delivery
2.10 Neonates/mothers who were visited by ASHA at home within 24 hours of delivery (in case of home delivery) or within 24 hours of return to home from hospital in case of hospital delivery
2.11 Neonates/mothers who received the recommended number of postnatal visits and at recommended times within first month of delivery by ASHA
2.12 Mothers who received satisfactory counseling about caring for neonatal baby from ASHA during her home visits after last delivery
2.13 Neonates who were satisfactorily examined by ASHA during her home visits after last delivery
2.14 Mothers who adopted selected neonatal care practices during first month after delivery
2.15 Neonates who had complications within first month of last delivery and sought care from ASHA
Young infant outcomes. Proportion of:
2.16 Mother who exclusively breast fed infant for first 6 months
2.17 Infants (6–8 months of age) who received solid, semi-solid, or soft foods during the previous day
2.18 Infants (6–8 months) who had received all three doses of pentavalent vaccine
2.19 Infants (6–8 months) who had diarrhea within last 2 weeks and received ORS from ASHA
2.20 Infants (6–8 months) with ARI/fever within last 2 weeks and family sought care from ASHA
2.21 Any harm suffered due to treatment or advice provided by the ASHA
Selected process indicators
2.22 Proportion of days ASHAs and medical officers logged in ImTeCHO mobile phone and web-based application respectively (login rate)
2.23 Proportion of scheduled tasks completed by ASHAs and medical officers in ImTeCHO mobile phone and web-based application respectively (task completion rate)
2.24 Number of pregnancies registered using mobile phones against expected number of registration
2.25 Number of complicated maternal, newborn, and child cases identified against expected
2.26 Stock-out rate (proportion of times when a drug or equipment was not available when required, e.g., non-availability of antibiotics in case of child with pneumonia)

### Control arm (comparator)

The control area will continue to receive usual health services from the government and other providers. All ASHAs in the control and intervention area will receive the refreshers' training described above.

### Sample size calculation

Based on the information available for a sample of the study area before baseline and earlier surveys, we assumed there will be 25 ASHAs (cluster size) in a PHC and the MACCI will be 36%. In the absence of existing information regarding the intra-class correlation, we assumed the intra-class correlation coefficient (ICC) to be 0.02. Assuming a loss of one cluster per arm and three ASHAs per PHC, for detecting 15% absolute improvement in the MACCI in the intervention arm compared to the control arm at the endline survey with 80% power and a 5% two-sided significance level, we estimated the required sample size per arm to be 11 PHCs/clusters. Similarly, we assumed that 46% of neonates/mothers would receive at least two postnatal home visits within the first week of delivery by the ASHA in the control arm based on earlier surveys. Assuming the loss of one cluster per arm and three ASHAs per PHC, we estimated the required sample size per arm to be six PHCs/clusters for detecting 20% absolute improvement in the proportion of neonates/mothers who received at least two postnatal home visits within the first week of delivery by the ASHA in the intervention arm compared to the control arm at the endline survey with 80% power and a 5% two-sided significance level.

### Randomization

**Fig. 2:**
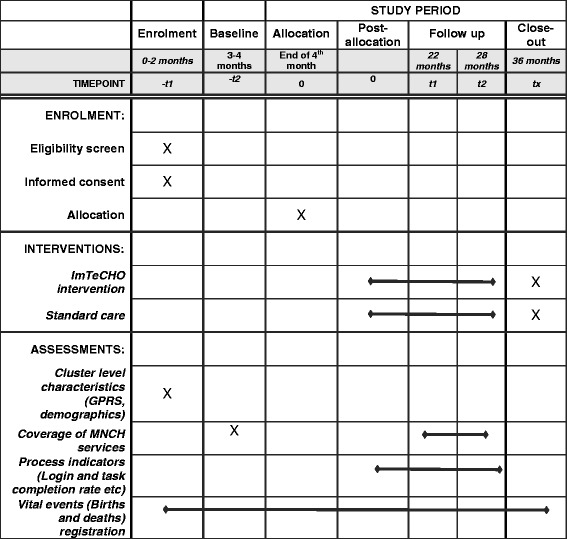
SPIRIT diagram for the study protocol

**Fig. 3:**
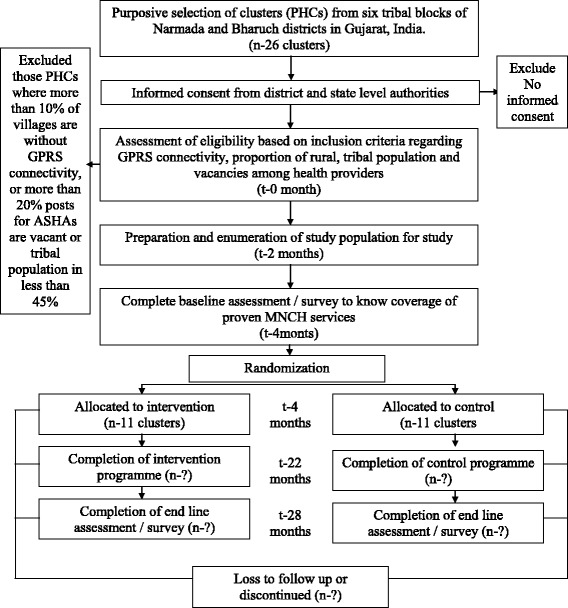
CONSORT study flow chart

Stratification was done to ensure that the prevalence of primary outcomes would be similar in the intervention and control groups along with almost equal selection of PHCs from the most backward Dediyapada block. The randomization was done after a baseline survey. The allocation ratio was 1:1. The stratified randomization was performed by a statistician at All India Institute of Medical Sciences (AIIMS, New Delhi) who was not involved in implementation of the intervention. The baseline variable used for stratification was the primary outcome indicator; the assessment will be described in detail in a later section. The mean value of the primary outcome indicator was used as a cut-off to divide each PHC into one of the two strata. The PHCs in each of the two strata were randomly allocated into an intervention and a control group. The randomization was done using the software *nQuery*. Due to the nature of the community-based intervention, blinding was not possible. See the Standard Protocol Items: Recommendations for Interventional Trials (SPIRIT) diagram (Fig. 2), the Consolidated Standards of Reporting Trials (CONSORT) study flow chart (Fig. 3), and the SPIRIT checklist (Additional file 1). Additional file 2 is a copy of the information sheet and consent form. Additional file 3 shows the statistical analysis plan.

### Enrollment of clusters

All clusters (PHCs) belonging to six high-priority tribal blocks of Bharuch and Narmada districts in Gujarat (except those where ImTeCHO is being implemented already as part of another project) having a 100% rural population and predominantly scheduled tribe population (at least 45%) were eligible to be included. Bharuch and Narmada districts were selected because both are predominantly tribal districts with several hard-to-reach, high-priority areas identified by the state government and with proximity to the lead organization conducting this study. Those PHCs were excluded where all medical officer posts and more than 20% of posts for ASHAs were vacant at the time of initiation of the study. PHCs that had more than 10% villages without GPRS (mobile connectivity) most of the time were excluded. Although the ImTeCHO mobile application can function without a GPRS signal, the lack of this signal in large areas of intervention will affect components of the intervention to a significant extent.

### Recruitment of respondents, measurement of outcomes, and follow-up

Household surveys will be used for assessing the above-mentioned outcomes of interest. The household survey tools are based on the District Level Household and Facility Survey (DLHFS-4) [27]. Table 3 briefly describes the measurement of outcomes.

**Table 3:** Measurement of outcomes

What	Where	When	How	By whom
Coverage of proven maternal and neonatal interventions, coverage of care among complicated maternal and neonatal cases	At household level in community	2 times: pre-intervention (baseline), post-intervention (endline)	Household survey as reported by Type-A respondents (mothers of infants aged 1–4 months)^a^	Data collection team
Coverage of proven young infant health interventions, coverage of care among complicated young infant cases	At household level in community	2 times: pre-intervention (baseline), post-intervention (endline)	Household survey as reported by Type-B respondents (mothers of infants aged 6–9 months)	Data collection team
Adherence to intervention, support, and supervision received by ASHAs	From ImTeCHO program data (online)	Post-intervention (endline)	From web interface and log of ImTeCHO facilitator	Investigators

^a^Mothers of infants aged 6–9 months surveyed at baseline

All pregnancy registrations, their outcomes, and any neonatal and infant deaths in all clusters throughout the study period will be counted as part of the ongoing pregnancy and mortality surveillance by a separate team of data collectors. Subsequently, all respondents who will be eligible during the survey period will be enrolled for a household survey using information from the ongoing pregnancy registration and mortality surveillance, whether or not they received the intervention. The data collection team will enter data into smartphones which will be loaded with a data collection tool with built-in checks for missing data and logical inconsistency. Data will be uploaded to a central server using the GPRS. A data administrator will check for any inaccuracies, and these will be clarified during a review meeting with the data collectors. Five percent of households will be randomly selected and re-interviewed by an independent quality assurance team using a truncated questionnaire.

### Timeline and duration of the study

This study is of 3 years’ duration from March 2015 to February 2018. Table 4 shows the timeline for the study. The development of the ImTeCHO intervention and formative evaluation is already completed and documented [15].

**Table 4:**
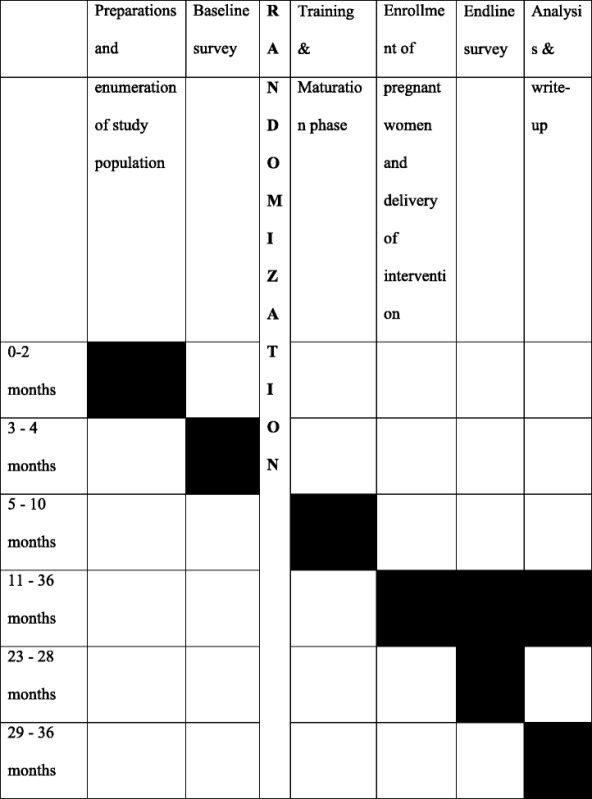
Timeline for the study

### Administrative structure

The implementation will be delivered by an implementation team, and evaluation will be done by an evaluation team; the teams will work independently of each other. Both teams are supervised by the principal investigator through meetings held twice a month. An advisory committee consisting of independent researchers, technical experts, academicians, and government administrators meets on a regular basis.

#### Implementation team

The implementation team consists of:

- Government staff which includes ASHAs, and PHC staff
- A program manager and program associate from SEWA Rural who supervise and support ImTeCHO facilitators and the helpline counselor, intervene in emergency situations, and also coordinate with medical officers and higher level government health officials
- Software developers from Argusoft India Ltd. who maintain the ImTeCHO software

#### Evaluation team

The evaluation team consists of:

- Data collectors who conduct surveillance activities to record vital events
- Supervisors who supervise the data collection team
- A data manager and statistician
- A research coordinator and a research associate from SEWA Rural who plan, execute, and oversee the evaluation efforts

### Data Safety and Monitoring Board

The Data Safety and Monitoring Board comprises independent scientists, ethicists, community members, and a statistician, and they meet on a regular basis. As the outcome measurements will be done at baseline and endline only, there is no plan to do an interim analysis. There is no plan to audit the conduct of the trial.

### Analysis plan

Data analysis will be done as per the statistical analysis plan (see Additional file 3). The primary analysis will be intention to treat, and the secondary analysis would be per protocol. Data will be analyzed taking into account cluster randomization. In case of any imbalance in any of the characteristics at the cluster or at the beneficiary level, adjustments will be done using the generalized estimating equation approach. For each of the primary and secondary outcomes, the effect size (95% confidence interval), after adjustment if required, would be computed. Each outcome will be compared between the intervention and control groups at the endline. Additionally, the difference in differences technique will be used for those outcomes which were measured at baseline and endline. A before and after analysis will also be done. STATA 13.0 will be used for data analysis. The results will be presented as per the statistical analysis plan.

### Ethics approval and consent to participate

The Multi-institutional Ethics Committee (Mumbai), Institutional Ethics Committee of SEWA Rural, and Ethics Review Committee of the World Health Organization (Protocol ID number: MCA00615) have approved the study. Informed consent will be obtained by trained data collectors from all respondents prior to a household survey. A copy of the information sheet and consent form can be found in Additional file 2. All personally identifiable information will be removed, and unique numbers will be assigned to the participants. Only the investigators will have access to data linking personally identifiable information with the unique numbers. The proposed application will contain medical information of individuals and hence demands high levels of data security. In view of this, the application shall accept, transmit, process, and store data using prudent security and data encryption practices. The database will be protected by a password. The study is registered at the Clinical Trial Registry of India (http://www.ctri.nic.in/Clinicaltrials/pdf_generate.php?trialid=11820&EncHid=&modid=&compid=%27,%2711820det%27), and the registration number for the submitted application is CTRI/2015/06/005847. We will disseminate findings of the study with all stakeholders through various strategies, including meetings and printed and visual media and publications. The final report will follow the CONSORT 2010 guidelines and its extension for cluster trials published in 2012 and non-pharmacologic treatment interventions [28].

### Problems anticipated

We need to assess the risk of some unplanned events during the course of the study which can have an impact on it. Some of the possibilities are:

- New schemes from the government such as starting a hotline, a voice messaging system, or another mHealth intervention, etc.
- A new service provider/non-governmental organization (NGO) starting to serve either the control or intervention area, which can impact outcomes
- New regulations regarding the use of mobiles from the government
- Major staffing issues at PHCs, which can impact outcomes

If there is a major reorganization of the PHCs by the government, then dropout of selected PHCs might be inevitable. Appropriate decisions will be taken on a case-by-case basis, and records will be maintained to document actions taken.

SEWA Rural is closely working with the government and other stakeholders to mitigate or minimize these and other unanticipated risks while preserving ethical and moral values.

### Organizations involved in this study

SEWA Rural is the lead organization. The Department of Health and Family Welfare, Government of Gujarat is implementing the intervention within the primary health care system with active facilitation by SEWA Rural. Argusoft India Ltd. and SEWA Rural are leading efforts towards development and maintenance of the ImTeCHO software.

### Nested studies

A few nested studies are being planned during the course of the trial as follows:

- Examine the effect of ImTeCHO intervention in the form of job aid to ASHAs, ANMs, medical officers, and PHC staff to reduce neonatal mortality rate, infant mortality rate, and undernutrition in tribal areas of Gujarat
- Examine cost-effectiveness of ImTeCHO intervention in the form of job aid to ASHAs, ANMs, and medical officers to increase coverage of selected MNCH interventions and care to be provided for complicated maternal, neonatal, and child cases in tribal areas of Gujarat
- Improve the coverage of proven interventions for caring of neonates with low birth weight using an innovative mHealth intervention
- Examine the effectiveness of mHealth intervention on skills and knowledge of ASHAs
- Compare the coverage of proven MNCH services between areas using ImTeCHO within a government health system and a voluntary organization

## Discussion

Insufficient evidence regarding effectiveness in improving outcomes, cost-effectiveness, and feasibility has been one of the most important barriers for scaling mHealth solutions [29]. This study will help answer some critical questions about the effectiveness and feasibility of implementing mHealth solution in area of MNCH. Reducing maternal, neonatal, and infant mortality is one of the Sustainable Development Goals [30]. If ImTeCHO is found to be effective and feasible, then investment of resources at a scale for replicating this model can be justified. More research after appropriate modification to the proposed intervention will be advised if the intervention is found to be not effective and/or not feasible in real-life situations. Some of the specific areas of impact are listed below. The web interface can be easily integrated into existing medical information systems. This study and its deliverables will provide an exact road map to be followed for implementing mHealth solutions for community-based MNCH. Deliverables of this study such as mobile phone applications, implementation plan, training modules, and process indicators can be adapted in other states immediately for wider use, as these will be based on existing policies and the health system in place. The endline survey will begin in the summer of 2017, and results will be available by the end of 2017.

### Trial status

The clusters were randomized in July 2015. The intervention has been implemented since February 2016 after completion of training of the ImTeCHO users.

## Additional files

Additional file 1: SPIRIT checklist. (DOC 97 kb)

Additional file 2: Information sheet and consent form. (DOC 35 kb)

Additional file 3: Statistical analysis plan. (DOC 267 kb)

## Abbreviations

ANC: Antenatal care
ANCS: Antenatal care service
ANM: Auxiliary Nurse Midwife
ASHA: Accredited Social Health Activist
AYUSH: Ayurveda, Yoga and Naturopathy, Unani, Siddha, and Homoeopathy
BCG: Bacillus Calmette-Guérin
CCI: Composite coverage index
DLHFS: District Level Household and Facility Survey
DPT: Diphtheria, pertussis and tetanus
EBF: Exclusive breast feeding for 6 months
FD: Facility delivery
GPRS: General Packet Radio Service
ICMR: Indian Council of Medical Research
IFA: Iron-folic acid
Inj.TT: Tetanus toxoid injection
MACCI: Modified ASHA-centric composite coverage index
MNCH: Maternal, neonatal, and child health
MSL: Measles vaccination
NGO: Non-governmental organization
NRHM: National Rural Health Mission
PHC: Primary Health Center
PNC: Postnatal care
SBA: Skilled birth attendance
SC: Scheduled caste
SEWA Rural: Society for Education, Welfare and Action - Rural
SMS: Short Message Service
ST: Scheduled tribe
URL: Uniform Resource Locator
VHND: Village Health and Nutrition Day
WHO: World Health Organization
