# Supplementary material for: Cluster randomized trial of a mHealth intervention “ImTeCHO” to improve delivery of proven maternal, neonatal, and child care interventions through community-based Accredited Social Health Activists (ASHAs) by enhancing their motivation and strengthening supervision in tribal areas of Gujarat, India: study protocol for a randomized controlled trial
Source: Trials. 2017 Jun 9;18:270. doi: 10.1186/s13063-017-1998-0 (PMC5466719; doi:10.1186/s13063-017-1998-0)
Supplement: Supplementary file 2 — Information sheet and consent form. (DOC 35 kb) [file 13063_2017_1998_MOESM2_ESM.doc]

Additional file 2: Information sheet and informed consent form

**Information sheet for study titled “Cluster Randomized Trial of a mHealth Intervention “ImTeCHO” to Improve Delivery of Proven Maternal, Newborn and Child Care Interventions through Community Based Accredited Social Health Activists (ASHAs) by Enhancing Their Motivation and Strengthening Supervision in Tribal Areas of Gujarat, India”**

This information sheet is to inform the health workers of the study area regarding objectives and their role for above mentioned study.

Namaste.

**Introduction**

We are researchers from SEWA Rural. We want to inform you about the objectives of above stated study which aims to evaluate use of mHealth Solutions (mobile phone technology) to improve delivery of proven maternal newborn and child care interventions through community based accredited social health activists (ASHAs) by enhancing their motivation and strengthening supervision in tribal areas of Gujarat, India.

**Why are we doing this study?**

The Government of India has appointed ASHA workers in every village under the National Rural Health Mission. One of the primary goal of ASHA program is to improve maternal, newborn and child health in rural areas. This study is being conducted to evaluate use of mobile phone technology to improve services provided by ASHAs.

**What is ImTeCHO intervention?**

The ImTeCHO is a mobile phone application to empower ASHAs and PHC staff. The application provides support to the ASHAs and PHC staff to improve community based maternal, newborn and child health services. ASHAs will be given mobile phones loaded with newly designed application to improve counseling, and management of sicknesses.The ImTeCHO application is currently use in more than 200 villages of Gujarat. Its use has resulted in positive effect on the health of mothers and children. So far, no serious risks have been noted during its use.

**What is expected of you in this study? (Type of Research Intervention and procedures)**

To test the effectiveness of the intervention, health workers in half of the study area would be requested to use mobile and web based application after receiving training; the remaining health workers will continue providing routine care. The ImTeCHO mobile phone application will provide the health workers guidance to carry out their routine work; however, the health worker should use their own discretion while taking final decision for managing the beneficiary based on her/his knowledge and overall assessment of the condition. We will request all the health workers to receive refresher’s training. At the end of study, we will interview mothers of infants from the study villagesabout events and services received by them during their last pregnancy, delivery and period immediately after delivery. We will share findings of the study with wider scientific community, health workers, local community, health administrators and policy makers through vernacular language newspapers, meetings, publications in scientific journals, presentations at various meetings and SEWA Rural’s community advisory board. We assure you that the findings of the survey will be only used to assess effectiveness of the ImTeCHO application; it will not be used to assess you at professional level and will not have any negative repercussions for your current job or future prospects.

**What are some potential benefits, risks and harm?**

During the study, health workers in the study area will be trained so that they can provide better services. If the ImTeCHO application is successful then it would minimize few important problems faced by ASHAs such as delay in payment of monetary incentives and drug stock outs. If use of mobile phone technology is found to be effective to improve services provided by ASHAs, than such technology might be used in other areas of Gujarat. Eventually, many other health workers will be potentially benefited. You will not be subjected to any harm by participating in this study.

**Permission from the health ministry**

We have received required permission from the Commissioner of Health, Department of Health and Family Welfare for the health workers to participate in the study.

This proposal has been reviewed and approved by the multi institutional ethics committee, Mumbai, which is a committee whose task it is to make sure that research participants are protected from harm. It has also been reviewed by the Ethics Review Committee of the World Health Organization (WHO), which is supporting the study.

If you have any questions, you can contact Dr. Pankaj Shah from SEWA Rural at following address.

Dr.Pankaj Shah,

**Principal Investigator,**

SEWA Rural, Jhagadia,

Dist: Bharuch, Gujarat,

PIN: 393110.

Ph: (02645)220021

With regards,

Dr. Pankaj Shah

**PART 2: Certificate of Consent**

1. I have received information sheet and appropriate answers to my questions.
2. I have been given information about goals and methods of the study.
3. I know that I can refuse to participate in the study at any time and information provided by me will remain confidential.
4. I know that my identity will remain confidential and personally identifiable information will not be published.
5. I know that I can contact at above address in case I have any questions.

I voluntarily consent to participate in this study.

Signature or thumb impression of participant: ..................................

Name of participant: ............................................

Date: ...................................

**Statement by the researcher/person taking consent**

I have accurately read out the information sheet to the potential participant, and to the best of my ability made sure that the participant understands that the following will be done:

1. We will ask questions about events and services received by you during your last pregnancy, delivery and post-partum period.

2. We will note responses on a digital form

3. We will publish findings of the study.

I confirm that the participant was given an opportunity to ask questions about the study, and all the questions asked by the participant have been answered correctly and to the best of my ability. I confirm that the individual has not been coerced into giving consent, and the consent has been given freely and voluntarily.

A copy of this ICF has been provided to the participant.

Print Name of Researcher/person taking the consent________________________

Signature of Researcher /person taking the consent__________________________

Date ___________________________
