# Supplementary material for: Cluster randomized trial of a mHealth intervention “ImTeCHO” to improve delivery of proven maternal, neonatal, and child care interventions through community-based Accredited Social Health Activists (ASHAs) by enhancing their motivation and strengthening supervision in tribal areas of Gujarat, India: study protocol for a randomized controlled trial
Source: Trials. 2017 Jun 9;18:270. doi: 10.1186/s13063-017-1998-0 (PMC5466719; doi:10.1186/s13063-017-1998-0)
Supplement: Supplementary file 3 — Statistical analysis plan. (DOC 267 kb) [file 13063_2017_1998_MOESM3_ESM.doc]

# STATISTICAL ANALYSIS PLAN FOR TRIAL

# “Cluster Randomized Trial of a mHealth Intervention “*ImTeCHO*” to Improve Delivery of Proven Maternal, Newborn and Child Care Interventions through Community Based Accredited Social Health Activists (ASHAs) by Enhancing Their Motivation and Strengthening Supervision in Tribal Areas of Gujarat, India”

**CTRI registration number:** CTRI/2015/06/005847

**Name of intervention: ImTeCHO (Innovative Mobile-phone Technology for Community Health Operations)**

**Prepared by: Investigators and Prof. R.M.Pandey (Head of department, Biostatistics, AIIMS, New Delhi, India)**

**Primary Sponsor: The Indian Council of Medical Research**

Table of Contents

[1 Introduction 1](#__RefHeading___Toc424833847)

[1 Primary Objective 1](#__RefHeading___Toc424833848)

[2 Secondary objectives 1](#__RefHeading___Toc424833849)

[3 Research Questions 2](#__RefHeading___Toc424833850)

[3.1 Primary research questions 2](#__RefHeading___Toc424833851)

[3.2 Secondary Research Questions 2](#__RefHeading___Toc424833852)

[4 Study description 3](#__RefHeading___Toc424833853)

[4.1 Study design 3](#__RefHeading___Toc424833854)

[4.2 Sample size 3](#__RefHeading___Toc424833855)

[4.3 Study regimens 6](#__RefHeading___Toc424833856)

[4.3.1 Intervention group 6](#__RefHeading___Toc424833857)

[4.3.2 Control group 6](#__RefHeading___Toc424833858)

[4.4 Study time points 6](#__RefHeading___Toc424833859)

[4.5 Inclusion and exclusion criteria 7](#__RefHeading___Toc424833860)

[5 Definition of Primary Outcome Indicators 9](#__RefHeading___Toc424833861)

[6 Process indicators 11](#__RefHeading___Toc424833862)

[7 Study population 12](#__RefHeading___Toc424833863)

[7.1 Intent to Treat Population (ITT Population) 12](#__RefHeading___Toc424833864)

[7.2 Per Protocol Population (PP Population) 12](#__RefHeading___Toc424833865)

[8 Statistical analysis 12](#__RefHeading___Toc424833866)

[8.1 General statistical methodology 12](#__RefHeading___Toc424833867)

[8.2 Eligibility for cases to be analyzed 12](#__RefHeading___Toc424833868)

[8.3 Characteristics of study population 13](#__RefHeading___Toc424833869)

[8.4 Description of Analysis 13](#__RefHeading___Toc424833870)

[8.5 Interim analysis 13](#__RefHeading___Toc424833871)

[8.6 Handling of withdrawn clusters 13](#__RefHeading___Toc424833872)

[8.7 Key Indicators 14](#__RefHeading___Toc424833873)

[8.8 Tabulation 14](#__RefHeading___Toc424833874)

[8.9 Tabulation at baseline 16](#__RefHeading___Toc424833875)

[8.10 Dummy tabulations at end line 19](#__RefHeading___Toc424833876)

# Introduction

To facilitate delivery of proven maternal and newborn interventions, a new cadre of village-based Community Health Workers, called Accredited Social Health Activist (ASHA), was created in 2005 under the aegis of the National Rural Health Mission in India. Evaluations have noted that coverage of selected maternal, newborn and child health (MNCH) services, to be delivered by ASHAs is low. Reasons for low coverage are inadequate supervision and support to ASHAs apart from insufficient skills, poor quality of training, and complexity of tasks to be performed.

Proposed study aims to implement, and evaluate an innovative intervention based on mobile-phone technology to improve performance of ASHAs through better supervision and support in predominantly tribal and rural communities of Gujarat, India. The intervention which is a newly-built mobile-phone application will be used and evaluated in three ways: (1) Mobile-phone as a job-aid to ASHAs to increase coverage of maternal and newborn care services (2) Mobile-phone as a job-aid to ASHAs and Auxiliary Nurse Midwives (ANMs) to increase coverage of care among maternal and newborn cases with complications by facilitating referral, if indicated and home-based-care (3) Web-interface as job-aid for medical-officers to improve supervision and support to ASHA program.

This will be a two-arm cluster randomized trial of 36 months duration. There will be eleven Primary Health Centers (with a population of approximately 20,000 populations each) in each arm. Primary outcome measures include coverage of selected MNCH services and care received by complicated cases. Outcomes will be measured by conducting household surveys at baseline, and post-intervention which will be compared with usual practice in control area where current level of services provided by the government will continue.

# Primary Objective

To Examine effect of mHealth solutions in the form of job-aid to ASHAs during her scheduled home visit to increase coverage of selected MNCH interventions to be provided by her in tribal and rural areas of Gujarat.

# Secondary objectives

- Examine effect of ImTeCHO intervention in the form of job aid to ASHA and ANM to increase coverage of care among complicated maternal, newborn and child cases by facilitating referral to a health facility and managing at home for those cases that unable to get referred in tribal areas of Gujarat.
- Examine effect of ImTeCHO intervention in the form of a job aid to medical officers and PHC staff to improve support and supervision of ASHAs.
- Examine process indicators to understand processes and level of adherence to intervention.
- Examine effect of ImTeCHO intervention in the form of job-aid to ASHAs, ANMs, medical officers and PHC staff to increase coverage of selected MNCH interventions and care to be provided for complicated maternal, newborn and child cases in tribal areas of Gujarat.

# Research Questions

## Primary research questions

Can mHealth solutions in the form of job-aid to ASHAs during her scheduled home visit increase coverage of following indicators compared with usual care among tribal areas of Gujarat?

## Secondary Research Questions

1. Can ImTeCHO intervention in the form of job-aid to ASHAs and ANM increase coverage of care among complicated maternal, newborn and child cases in tribal areas of Gujarat?
2. Can ImTeCHO intervention in the form of a job aid to medical officers and PHC staff improve support and supervision of ASHAs?
3. Examine process indicators to understand processes and level of adherence to intervention.
4. Can ImTeCHO intervention in the form of job-aid to ASHAs, ANMs and medical officers to increase coverage of selected MNCH interventions and care to be provided for complicated maternal, newborn and child cases in tribal areas of Gujarat cost-effective?

# Study description

## Study design

This will be a two arm, parallel, stratified cluster randomized trial in which unit of randomization will be a PHC. Stratification is required to ensure that prevalence of primary outcomes are similar in the intervention and control groups along with almost equal selection of PHCs from from the most backward Dediyapada block. Stratification will be also done to improve power and precision. The randomization will be done after baseline survey. Along with primary outcomes, randomization would help to balance cluster size across both groups. The allocation ratio will be 1:1.

## Sample size

Please see Table 1 for sample size calculation and associated assumptions. Based on assumptions listed in the Table 9, eleven clusters (PHCs) will be randomized in each arm with which we will be able to answer the above mentioned questions. The assumptions used for sample size calculation are based on data collected by SEWA Rural in a sample of study area and current area of pilot implementation along with other relevant wide scale surveys by the government. However, there is no existing data available from the whole study area about primary outcome of interest. Hence, sample size will need to be revised based on findings of baseline survey and definition of “success” if large discrepancy is found between current assumptions and baseline survey findings. Considering required sample size of 11 PHCs each in intervention and control area, six blocks (total 26 PHCs, out of which 23 meet all eligibility criteria) listed above should suffice to select study clusters.

As intervention is primarily affecting ASHAs, analysis will be done at ASHA level. Therefore, number of ASHAs per PHC will be used as “cluster size”. Based on indirect information before baseline, we assumed there will be 25 ASHAs in a PHC and MACCI of 36%. In absence of existing information regarding the intraclass correlation (ICC), we will assume ICC to be 0.02. Assuming loss of one cluster per arm and three ASHAs per PHC, for detecting 15% absolute improvement in MACCI in intervention arm compared to control arm at endline survey with 80% power and 5% two-sided significance level, we estimated that required sample size per arm will be 11 PHCs/clusters. Similarly, we assumed 46% of neonates/mothers would receive at least two postnatal home visits within first week of delivery by ASHA in control arm. Assuming loss of one cluster per arm and three ASHAs per PHC, we estimated that required sample size per arm will be six PHCs/clusters for detecting 20% absolute improvement in proportion of neonates/mothers who received at least two postnatal home visits within first week of delivery by ASHA in intervention arm compared to control arm at endline survey with 80% power and 5% two-sided significance level..

## Study regimens

### Intervention group

Complete detail of the intervention is included in study protocol. Following are major components of the intervention

1. **ImTeCHO mobile phone and web application having following objectives**

- **Mobile phone as job aid to ASHA to increase coverage MNCH care**
- **Mobile phone as job aid to ASHA and ANM to facilitate care for mother, newborn and child with complications**
- **Web interface to provide timely information to medical officer to facilitate monitoring and supporting program**

1. **Help line/telephone care from SEWA Rural**
2. **mHealth facilitators from SEWA Rural (one for every two to three PHCs)**
3. **Training, and mentoring for use of mobile phone and refresher training for module 6 and 7**
4. **Project team at SEWA Rural and district health societies**

### Control group

The control area will continue to receive usual health services from government and other providers. All ASHAs in control and intervention area will be trained to get up-to-date to provide recommended maternal, newborn and child care. ASHAs will receive refreshers’ training based on ASHA module-6 and 7 which provides skills for the maternal care services that she is expected to deliver including counselling pregnant women, ensuring complete antenatal care through home visits and enabling care at monthly Village Health and Nutrition Days (VHND), assisting households to make birth plan and supporting households for safe delivery. Regarding newborn care, ASHAs will be trained to undertake at least 6 post-partum visits, counsel and problem solve on breastfeeding, keep the baby warm and identify and do basic management of LBW (Low Birth Weight) and pre-term baby, perform examinations needed for identification/first contract care for sepsis and asphyxia. Size of control area will be same as intervention area (11 PHCs).

## Study time points

There will be 3 phases:

1. Phase 1 (Preparations): The recruitment and training of project staff along with revisions in protocol based on technical help from the experts from the ICMR and WHO will be done.
2. Phase 2 (Baseline data collection- 0-2 months): Baseline data will be collected from intervention and control area. Findings will be used for randomization to be done at the end of this phase.
3. *Phase 3 (Training and maturation phase- 5 - 10 months):* All ASHAs (approximately 250 in number) and PHC staff will be trained to use mobile phone and web interface respectively to implement intervention. After training, ASHAs will require extra support for initial weeks to get them used to working with mobile phones and initial technical troubleshooting. Based on SEWA Rural’s recent experience with introduction of mobile phone technology, it is essential to have adequate time period for maturation phase so that intervention become fully functional at the end of the maturation phase. All the ASHAs from control area will receive refreshers’ training during this period as well. The PHC staff will be trained for using web and mobile based application.
4. *Phase 4- Intervention implementation- 11-36 months):* At village level, existing ASHAs within the government set-up, who will be trained during the maturation phase, will use mobile phone to implement the intervention. Medical officer and PHC staff will use the web interface to monitor and support program.
5. Phase 5 (Endline data collection- 23-28 months):Endline data collection from intervention and control area will take place during this phase.
6. *Phase 6 (analysis and report writing- 28-36 months):* Data will be analyzed and complete report will be completed.
7. *Phase 7(ongoing surveillance-* 0 to 36 months): Ongoing surveillance for pregnancy registration and death will be done throughout the study period.

Table 10 Phase wise deployment of intervention

|  | Preparations | Baseline survey | **R**  **A**  **N**  **D**  **O**  **M**  **I**  **Z**  **A**  **T**  **I**  **O**  **N** | Training & Maturation phase | Intervention Implementation | Endline survey | Analysis & write-up |
| --- | --- | --- | --- | --- | --- | --- | --- |
|
|
|
|
|
|
| 0-2 months |  |  |  |  |  |  |
| 3 - 4 months |  |  |  |  |  |  |
| 5 - 10 months |  |  |  |  |  |  |
| 11 - 36 months |  |  |  |  |  |  |
| 23-28 months |  |  |  |  |  |  |
| 29 - 36 months |  |  |  |  |  |  |

## Inclusion and exclusion criteria

All clusters (PHCs) belonging to Valia, Netrang (except those where ImTeCHO is being implemented already as part of another project), Dediyapada, Nandod, Garudeshwar and Tilakwada blocks with 100% rural population and scheduled tribe population of more than 45% will be eligible to be included. Those PHCs will be excluded where all medical officer posts, and 20% post for ASHAs are vacant at the time of initiation of study. Those PHCs will be excluded whose more than 10% villages have no mobile signal most of the time. Although ImTeCHO mobile application can function without GPRS signal, lack of such signal in large areas of intervention will affect components of intervention to significant extent. PHCs where internet cannot be accessed reliably by medical officer and PHC staff to view web interface and an alternative arrangement is not possible will be excluded too as web interface is important part of the ImTeCHO intervention.

# Definition of Primary Outcome Indicators

The first being proportion of neonates/mothers who receive at least two postnatal home visits within first week of delivery by ASHA. As most of the neonatal deaths occur during first few days of life, it is recommended that neonates should be visited three times during first week including day of delivery, third day and preferably seventh day[[1]](#footnote-2). However, most (78% according to coverage evaluation survey of UNICEF in 2009) of the deliveries in Gujarat now occur in facility where ASHAs’ role ends up being limited in presence of the facility based more qualified health workers[[2]](#footnote-3). Also, ASHAs’ visit on the day of delivery is influenced by variety of factors with limited role of the ImTeCHO intervention. Hence, we decided to focus on coverage of ASHAs’ two visits during first week after the mother/neonates returns home after discharge from the facility.

- Improve proportion of neonates/mothers who received at least two postnatal home visits within first week of delivery by ASHA from 46% to 66%.

The second primary outcome of interest will be a composite coverage index will be calculated using following formula and rationale.

Modified ASHA-centric composite coverage index (MACCI) = 0·25 × (0.33 × [Complete ASHA home visit during antenatal period +Full ANCS+SBA] + [Complete HBNC] + 0·5 × [DPT3 + EBF] + 0·33 × [Care seeking for newborncomplications+ORT + ARI/febrile illness])

In which,

1. Maternal care domain

- Complete ASHA home visit during antenatal period = proportion of mothers who were visited at home by ASHA at least three times during last pregnancy including at least one visit during last trimester,
- Full ANCS = proportion of mothers with full antenatal examination (at least three antenatal examination, one Inj.TT and 100 IFA tablets)Error: Reference source not found
- SBA = proportion of mothers who delivered in a facility as most of the deliveries attended by skilled attendant are those taking place at a facility in GujaratError: Reference source not found

1. Newborn care domain

- Complete HBNC = proportion of neonates/mothers who received the recommended number of postnatal visits and at recommended times within first month of delivery by ASHAError: Reference source not found

1. Young infant care domain

- DPT3 = proportion of infants (6-8 months) who received three doses of diphtheria, pertussis, and tetanus vaccine or three doses of pentavalent vaccine
- EBF = proportion of infant (6-8 months) who were exclusively breast fed for first six months,

1. Morbidity management domain

- Care seeking for newborn complications= proportion of neonates who had complications within first month of last delivery and sought care from ASHA
- ORT = proportion of infants (6-8 months) who had diarrhea within last two weeks and received ORS from ASHA
- ARI/fever = proportion of infant (6-8 months) with ARI/fever within last two weeks and sought care from ASHA

The weight of morbidity domain will be nullified if no morbidity was found for a particular ASHA.

Improve MACCI from 36% to 51%.

Regarding the use of composite indicator, we were guided by Composite coverage index (CCI) which is now widely used to measure coverage of key MNCH interventions and strength of health-system[[3]](#footnote-4),[[4]](#footnote-5),[[5]](#footnote-6). It provides summary measure to assess continuum of care. Formula for calculating CCI is as following.

CCI= 0·25 × (FPS + 0.5 × [SBA + ANCS] + 0·25 × [2DPT3 + MSL + BCG] + 0·5 × [ORT + CPNM])Error: Reference source not found

In which FPS is family planning needs satisfied. Above interventions were selected because of its impact on mortality, measurability, availability of data, relevance with health system strength and as reflection across continuum of care. All above indicators were calculated using standard countdown 2015 definitions [[6]](#footnote-7).

For this study, a modified composite summary measure is required which can reflect (1) coverage of MNCH interventions to be provided by ASHA in India (2) scope of ImTeCHO intervention (3) relevance in India. Therefore, CCI was modified without violating basic concept of CCI. Based on evidence of effectiveness of postnatal home visits by ASHA to reduce neonatal mortality in India and national recommendations from the government, a measure of postnatal home visit was added in the modified MACCI. Also, post-natal care for newborns is a countdown 2015 indicatorError: Reference source not found. Considering high prevalence of malnutrition in India, and role of ASHA in promoting young infant feeding practices, exclusive breast feeding up to six months was included in MACCI. It is also a countdown 2015 indicators. Care seeking for newborn was included considering its important association with newborn mortality, role of ASHAs and ImTeCHO’s component for management complications. Management of ARI/febrile illness was added as it was done during DLHFS which is being used for this study as wellError: Reference source not found. Family planning needs satisfied (FPS) was removed as it FPS is not focus of ImTeCHO intervention. Coverage of measles was removed because the respondents for the endline survey will be limited to mothers of infants between ages of six to eight months. Also, coverage of measles and BCG vaccination is already highError: Reference source not found. Hence, formula for MACCI is designed as following:

Modified ASHA-centric composite coverage index (MACCI) = 0·25 × (0.33 × [Care Complete ASHA home visit during antenatal period +Full ANCS+SBA] + [Complete HBNC+] + 0·5 × [DPT3 + EBF] + 0·33 × [Care seeking for newborncomplications+ORT + ARI/febrile illness])

Same weight was given to each of the all four main domains of interventions throughout the continuum of care which includes maternal, newborn, young infant care and care seeking for complications.

# Process indicators

| *Intervention 1: Mobile as a job-aid to ASHAs during her scheduled home visit to increase coverage of selected MNCH interventions* | |
| --- | --- |
| 1.1 | ASHA attendance rate (login rate)* |
| 1.2 | Number of home visit forms filled using mobile phones against expected (Task completion rate)* |
| 1.3 | Number of pregnancy registration forms filled using mobile phones against expected number of registration |
| 1.4 | Time taken (Mean, median, range) to complete mobile based home visit forms (Rationale: indirect measure of quality of interview) |
| 1.5 | Proportion of live and still births reported on the day of outcome |
| 1.6 | Proportion of beneficiaries who attended VHND against expected number (VHND attendance rate) |
| 1.7 | Line listing of beneficiaries with various due services (eg. ANC examination, HBNC, Vaccination, growth monitoring, ) |
| *Intervention 2: Mobile phone as job aid to ASHA and ANM to increase coverage of care among complicated maternal, newborn and child cases by facilitating referral to a health facility and managing at home for those cases that refuse to get referred* | |
| 2.1 | Number of complicated maternal (severe anaemia), newborn (LBW) and child (severe underweight) cases identified against expected |
| *Intervention 3: mHealth solutions in form a web interface to provide tools and timely information to PHC staff for monitoring and supporting MNCH program* | |
| 3.1 | Proportion of days when web interface was reviewed by medical officer (Attendance of medical officer with use of web interface) |
| 3.2 | Stock-out rate (Proportion of times when a drug or equipment was not available when required. Eg. Non-availability of antibiotics in case of of child with pneumonia) |
| 3.3 | Timeliness of incentive payment to ASHA |

# Study population

## Intent to Treat Population (ITT Population)

All eligible respondents belonging to clustersrandomized to the study groups*irrespective* of (1) whether they receive full course of study treatment or not and (2) major protocol deviations.

For child health interventions, all woman who are native of study village and is mother of an infant who is six to eight months old at the time of survey will be considered as eligible respondents for evaluation. For maternal and newborn health intervention, eligible respondents will be all women who are native of study village, and was in study cluster at the time of birth in case of home deliveries and in case of institutional deliveries if the she went to hospital from study cluster and came back to same study cluster immediately after delivery

## Per Protocol Population (PP Population)

All eligible respondents belonging to all clusters randomized and received the full course of the intervention with no major protocol deviations.

# Statistical analysis

## General statistical methodology

The continuous data will be summarised using number of respondents (N), mean, standard deviation (SD), median, minimum, maximum and interquartile range. The efficacy data will be summarised using frequencies (n) and percentages (%), difference in proportion, p value and confidence interval for difference in proportions. The categorical data will be summarised using frequencies (n) and percentages (%).

## Eligibility for cases to be analyzed

The analysis will only be done in cases which are eligible. Details of eligibility is mentioned in section 4.5.

## Characteristics of study population

The following basic characteristics will be assess

- Age of the mother
- Education
- Caste
- Parity
- Place of delivery

Infant mortality rates and neonatal mortality rates will be calculated for each of the areas.

## Description of Analysis

Data cleaning will be done using various relational checks and it will locked before analysis. Data analysis will be done as per the statistical analysis plan. Primary analysis will be intention to treat and secondary analysis would be per protocol. Data will be analysed taking in to account cluster randomization. Analysis would be done at various levels i.e. at ASHA level, PHC level, subcenter level and at the beneficiary level. In the analysis at ASHA/subcenter/PHC level, cluster would be the PHC while for analysis at beneficiary level; outcomes would be considered correlated within the PHC in block and among the beneficiary under a given ASHA. Before comparing the primary and secondary outcomes at the ASHA or at the beneficiary level, both cluster level and the unit of analysis level characteristics would be compared. In case of any imbalance in any of the characteristics at cluster or at the beneficiary level, both cluster level and the unit of analysis level characteristics would be compared. In case of any imbalance in any of the characteristics at cluster or at the beneficiary level, adjustments will be done using Generalized Estimating Equation approach. Categorical variables will be summarized using frequency (5) and quantitative variables will be summarized at mean/median and SD/IQR, as appropriate. For each of primary and secondary outcomes, effect size (95% confidence interval, after adjustment if required, would be computed. Each outcome will be compared between intervention and control group at endline. Additionally, difference in difference technique will be used for those outcomes which were measured at baseline and endline. Before and after analysis will be also done. STATA 13.0 will be used for data analysis. Results will be presented as per statistical analysis plan.

## Interim analysis

There will be no predetermined interim analysis, but will be performed on request by Data Safety Monitoring Committee or Steering Committee.

## Handling of withdrawn clusters

Clusters will be considered to have withdrawn from the study if they had entered into the study but did not complete the treatment throughout the study period. The information recorded for withdrawn clusters will be analyzed as obtained during measurement of outcome indicators during ongoing surveillance and endline survey in the intention to treat analysis.

## Key Indicators

**Maternal outcomes**

- Mothers with full antenatal checkup (at least three antenatal examination, one Inj.TT and 100 IFA tablets)
- Mothers who had 4 or more ANC examination by ANM/doctor including at least one examination in last trimester
- Mothers who were visited at home by ASHA at least three times during last pregnancy including at least one visit during last trimester
- Mothers who had institutional deliveries

**Neonatal outcomes**

- Neonates/mothers who were visited by ASHA at home within 24 hours of delivery (in case of home delivery) or within 24 hours of return to home from hospital in case of hospital delivery
- Neonates/mothers who received the recommended number of postnatal visits and at recommended times within first month of delivery by ASHA
- Neonates who had complications within first month of last delivery and sought care from ASHA

**Young child (6-8 months) outcomes**

- Mother who exclusively breastfed infant for first six months
- Infants 6–8 months of age who receive solid, semi-solid or soft foods during the previous day
- Infant (6-8 months) with ARI/fever within last two weeks and sought care from ASHA
- Infant (6-8 months) who had NOT received all three doses of pentavalent vaccine

## Tabulation

Finally the tabulation of the data as per the dummy tables provided will be done.The indicators will be calculated in the intervention and control areas, the differences will be tested using chi-square test. It will comprise of following domains.

- Socio-demographic characteristics of women
- Coverage of key MNCH interventions
- Selected cluster level characteristics in study area

## Tabulation at baseline

**Table 1A** Selected socio-demographic characteristics and coverage of key MNCH interventions among type-A respondents (mothers of infant whose age is from six to nine months) in study area at the time of baseline line survey

| **Characteristics** | **Number of respondents (%)** | |  |
| --- | --- | --- | --- |
|  | **Intervention area** | **Comparison area** | **p-value** |
|  |  |  |  |
| **Sociodemographic characteristics of women who had live birth during baseline year (2014)** | | | |
| **Number of mothers, n1** |  |  |  |
| Maternal age, mean (years) |  |  |  |
| Maternal education  No formal education  1-8 standard  >8^th^ standard |  |  |  |
|  |  |  |  |
| Para  1  2  =>3 |  |  |  |
|  |  |  |  |
| Maternal caste  Scheduled tribe  SC/OBC  Other |  |  |  |
|  |  |  |  |
| Place of delivery  Hospital  Home  On the way |  |  |  |
|  |  |  |  |
| **Coverage of key MNCH interventions among respondent type-A at baseline survey** | | | |
| **MACCI** |  |  |  |
| **Maternal and newborn health interventions, n** |  |  |  |
| Mothers who had full antenatal checkup* ( |  |  |  |

| Mothers who had 4 or more ANC examination by ANM/doctor including at least one examination in last trimester |  |  |  |
| --- | --- | --- | --- |
| Mothers who were visited at home by ASHA at least three times during last pregnancy including at least one visit during last trimester |  |  |  |
| Mothers who had institutional deliveries |  |  |  |
| Neonates/mothers who were visited by ASHA at home within 24 hours of delivery (in case of home delivery) or within 24 hours of return to home from hospital in case of hospital delivery |  |  |  |
| Neonates/mothers who received the at least two postnatal home visits within first week of delivery by ASHA |  |  |  |
| Neonates/mothers who received the recommended number of postnatal visits and at recommended times within first month of delivery by ASHA |  |  |  |
| Neonates who had complications within first month of last delivery and sought care from ASHA |  |  |  |
| **Child health interventions, n** |  |  |  |
| Mother who exclusively breastfed infant for first six months |  |  |  |
| Infants 6–8 months of age who receive solid, semi-solid or soft foods during the previous day |  |  |  |
| Infant (6-8 months) with ARI/fever within last two weeks and sought care from ASHA |  |  |  |
| Infants (6-8 months) with diarrhea within last two weeks and received ORS from ASHA |  |  |  |
| Infant (6-8 months) who had received all three doses of pentavalent vaccine |  |  |  |
| *at least three antenatal examination, one Inj.TT and 100 IFA tablets |  |  |  |

**Table 1B** Selected cluster level characteristics in study area at the time of baseline line survey

| **Characteristics** | **Number of respondents (%)** | |  |
| --- | --- | --- | --- |
|  | **Intervention area (11 PHCs)** | **Comparison area (11 PHCs)** | **p-value** |
|  |  |  |  |
| Average population of cluster |  |  |  |
| Proportion of ST (%) |  |  |  |
| Proportion of vacant ASHA posts |  |  |  |
| Proportion of vacant ANM posts |  |  |  |
| Proportion of PHCs without a medical officer (MBBS or AYUSH) |  |  |  |
| Proportion of PHCs with functional delivery facility |  |  |  |
| Proportion of villages which are not accessible by road during monsoon |  |  |  |

## Dummy tabulations at end line

**Table 2**Selected socio-demographic characteristics of type-A and type-B respondents (mothers of infant whose age is from six to nine months) in study area at the time of end line survey

| **Characteristics** | **Number of respondents (%)** | |  |
| --- | --- | --- | --- |
|  | **Intervention area (n=…….)** | **Comparison area**  **(n=…….)** | **p-value** |
| Maternal age |  |  |  |
| <=20 y |  |  |  |
| >20 |  |  |  |
| Maternal education |  |  |  |
| No formal education |  |  |  |
| 1-8 standard |  |  |  |
| >8^th^ standard |  |  |  |
| Gravida |  |  |  |
| 1 |  |  |  |
| 2 |  |  |  |
| =>3 |  |  |  |
| Maternal caste |  |  |  |
| Scheduled tribe |  |  |  |
| SC/OBC |  |  |  |
| Other |  |  |  |
|  |  |  |  |
|  |  |  |  |
|  |  |  |  |

**Table 3** Adherence to protocol (output indicators) by ASHA during their home visitations to type-A and type-B respondents during antenatal, postnatal and early childhood period

| **Indicator** | **Cluster mean** | |  |  |
| --- | --- | --- | --- | --- |
|  | **Intervention area (%)** | **Comparison area (%)** | **Unadjusted** Effect size(95% CI) | **Adjusted** Effect size(95% CI) |
| **Antenatal period** |  |  |  |  |
| At least 3 home visits by ASHA during last pregnancy (other than mobilizing for VHND) including at least one visit during last trimester |  |  |  |  |
| During home-visits, ASHA counseled about:  Satisfactory ANC counseling (at least 5 of below)  Birth planning and complication readiness  Conveyed expected date of delivery  Danger signs of pregnancy  examination by ANM/doctor  Institutional delivery  Early essential newborn care  Contacting ASHA if danger sign appears |  |  |  |  |
|  |  |  |  |  |
| ASHA visited at home within 24 hours of delivery (in case of home delivery) or within 24 hours of return to home from hospital in case of hospital delivery |  |  |  |  |
| At least two home visits by ASHA within first week after delivery |  |  |  |  |
| ASHA visited at home at least five times within first month after last delivery AND at least two of those visits were within first week after delivery* |  |  |  |  |
| ASHA counseled about following during her home visits within first month of delivery:    Satisfactory newborn care counseling (at least five of below)    Exclusive breast feeding  Proper attachment for breast feeding  Keeping baby warm by covering in cloth  Delaying first bath  Kangaroo mother care  Caring for umbilical cord  Washing hands before handling baby  Vaccination  Danger signs of newborn |  |  |  |  |
| ASHA performed following tasks on newborn during her home visits within first month of delivery  Satisfactory newborn examination(at least 3 of following)    Took temperature of newborn  Took weight of newborn  Examined skin and umbilicus  Washed her hands before examining  Observed breast feeding the baby in her presence |  |  |  |  |
|  |  |  |  |  |
|  |  |  |  |  |
| ASHA performed following nutritional counseling for young child within last three months  Initiate complementary food at six months  Adding oil, sugar and jiggery to food  Informed status of child on WHO growth chart (green, yellow or red) within last 3 months |  |  |  |  |
|  |  |  |  |  |
| Care seeking for young child  ASHA motivated to attend VHND within last 3 months  ASHA motivated mother to contact her in case the child suffers from diarrhea, fever or pneumonia |  |  |  |  |

*primary outcome indicator

**Table 4** Knowledge and practice (outcome indicators) among type-A and type-B respondents during antenatal, postnatal and early childhood period

| **Indicator** | **Cluster mean** | |  |  |
| --- | --- | --- | --- | --- |
|  | **Intervention area (% )** | **Comparison area (% )** | **Unadjusted** Effect size(95% CI) | **Adjusted** Effect size(95% CI) |
| **Antenatal period** | | | | |
| First antenatal examination by ANM/doctor during first trimester |  |  |  |  |
| Knowledge about danger signs of pregnancy  Able to state at least three danger signs |  |  |  |  |
| At least four antenatal check-up by ANM or doctor with at least one check up in last trimester |  |  |  |  |
| Received at least one dose of Inj.TT during last pregnancy |  |  |  |  |
| Consumed at least 100 Iron-FolicAcid (IFA) during pregnancy |  |  |  |  |
| **Delivery and neonatal period** | | | | |
| Delivery at hospital |  |  |  |  |
| Practice of newborn care immediately after delivery  Early initiation (within 1 hour) of breastfeeding  Colostrums was fed  Bathing delayed after first day  No prelacteal feed administered |  |  |  |  |
| Knowledge about danger signs of newborn  Able to correctly state at least 3 danger signs of newborn |  |  |  |  |
| Mother/family practiced following at home during first month after delivery  Kangaroo mother care  Did not apply anything on cord  Washed hands before handling baby |  |  |  |  |
| Young child related | | | | |
| Nutritional outcomes  Practiced exclusive breast feeding *until just under* 6 months of age  Child was taken to get weighted at least once during last 3 months  Child who received solid, semi-solid or soft foods during previous day  Child was fed solid, semisolid or soft food at least twice within last 24 hours (Minimum meal frequency)  Child was fed solid, semisolid or soft food with added oil, jiggery or sugar at least once during previous day  Mother knew status of child on WHO growth chart (green, yellow or red) |  |  |  |  |
| Health seeking  Visited ANM or doctor at least once within last 3 months (at VHND or any health facility)  Mother knew that she can contact ASHA for help in case child suffers from diarrhea, fever or pneumonia |  |  |  |  |
| Vaccination  Received all three doses of pentavalent vaccines |  |  |  |  |

**Table 5** Coverage of care for maternal, newborn and childhood complications among type-A respondents by study area

| Indicator | Cluster mean | |  |  |
| --- | --- | --- | --- | --- |
|  | Intervention area | Comparison area | Unadjusted Effect size(95% CI) | Adjusted Effect size(95% CI) |
| Antenatal maternal complications | | | | |
| Suffered from at least one antenatal complication during last pregnancy |  |  |  |  |
| Sought help from ASHA for antenatal complication |  |  |  |  |
| Suffered from at least one postnatal complication within six weeks of last delivery |  |  |  |  |
| Sought care from qualified health worker |  |  |  |  |
| Sought help from ASHA for postnatal complication |  |  |  |  |
| Mothers who suffered from at least one serious complications during last pregnancy or within six weeks of last delivery and sought care from a qualified health personnel |  |  |  |  |
| Newborn complications | | | | |
| Suffered from at least one newborn complication within two weeks of last delivery |  |  |  |  |
| Sought help from ASHA for newborn complication |  |  |  |  |
| Sought care from qualified health worker for newborn complications |  |  |  |  |
| Mothers who provided Kangaroo Mother Care (KMC) to their low birth weight babies within first month of last delivery |  |  |  |  |
| Q.384 (Q.83)Harmed by any medicine given by ASHA |  |  |  |  |
| Childhood complications | | | | |
| Suffered from diarrhea within last two weeks |  |  |  |  |
| Child received ORS |  |  |  |  |
| Child received ORS and breast feeding was continued |  |  |  |  |
| Child received ORS and ORS was supplied by ASHA |  |  |  |  |
| Suffered from pneumonia/fever within last one months |  |  |  |  |
| Child received ORS and Zinc |  |  |  |  |
| Sought help from ASHA for pneumonia/fever |  |  |  |  |

**Table 6 Gap in modified ASHA-centric composite coverage index among type-a and type-b respondents by study area**

**Modified ASHA-centric composite coverage index=** Modified ASHA-centric Composite Coverage Index =  0•25 × (0.33 × [Complete ASHA home visits during antenatal period +Full ANC + Skilled Birth Attendance] + [Complete Home Based Newborn Care] + 0•5 × [DPT3 + Exclusive Breast Feeding] + 0•33 × [Care seeking for newborn complications + ORS + Care seeking for pneumonia/febrile illness])

**Actual indicator would be gap in MACCI= 1 - MACCI**

| Indicator | Cluster mean (%) | | Effect size, (95% CI) | Adjusted Effect size(95% CI) |
| --- | --- | --- | --- | --- |
|  | Intervention area | Comparison area |  |  |
| MACCI |  |  |  |  |

**Table 7** A Process indicators/Trend of adherence to intervention (Input indicators) by ASHAs, PHC and SEWA Rural staff in intervention area over the course of study period

| **Sr no** | **Process indicators** |
| --- | --- |
| *Intervention 1: Mobile as a job-aid to ASHAs during her scheduled home visit to increase coverage of selected MNCH interventions* | |
| 1.1 | ASHA attendance rate (login rate)* |
| 1.2 | Number of home visit forms filled using mobile phones against expected (Task completion rate)* |
| 1.3 | Number of pregnancy registration forms filled using mobile phones against expected number of registration |
| 1.4 | Time taken (Mean, median, range) to complete mobile based home visit forms (Rationale: indirect measure of quality of interview) |
| 1.5 | Proportion of live and still births reported on the day of outcome |
| 1.6 | Proportion of beneficiaries who attended VHND against expected number (VHND attendance rate) |
| 1.7 | Line listing of beneficiaries with various due services (eg. ANC examination, HBNC, Vaccination, growth monitoring, ) |
| *Intervention 2: Mobile phone as job aid to ASHA and ANM to increase coverage of care among complicated maternal, newborn and child cases by facilitating referral to a health facility and managing at home for those cases that refuse to get referred* | |
| 2.1 | Number of complicated maternal (severe anaemia), newborn (LBW) and child (severe underweight) cases identified against expected |
| *Intervention 3: mHealth solutions in form a web interface to provide tools and timely information to PHC staff for monitoring and supporting MNCH program* | |
| 3.1 | Proportion of days when web interface was reviewed by medical officer (Attendance of medical officer with use of web interface) |
| 3.2 | Stock-out rate (Proportion of times when a drug or equipment was not available when required. Eg. Non-availability of antibiotics in case of of child with pneumonia) |
| 3.3 | Timeliness of incentive payment to ASHA |

*May check dose-response relationship , Source of data: ImTeCHO web interface throughout the study period

**Table 7** B Process indicators/Trend of adherence to intervention (Input indicators) by ASHAs, PHC and SEWA Rural staff in intervention area over the course of study period (Measuring efforts towards supervision, support and motivation)

| **Sr no** | **Process indicators** |
| --- | --- |
| 1. *mHealth solutions in form a web interface for better supervision of ASHAs* | |
| 1.1 | Proportion of ASHAs whose performance was reviewed by SEWA Rural’s ImTeCHO facilitator over a phone call per month |
| 1.2 | Proportion of ASHAs who were contacted by ImTeCHO facilitator (mostly through phone) every month for non-adherence to intervention |
| 1.3 | Proportion of ASHAs who were visited in field by ImTeCHO facilitator for quality improvement |
| 1.4 | Proportion of PHC meetings when[data from] ImTeCHO was used at least once for supervision by PHC staff |
| 1.5 | Medical officer’s login and task completion rate |
| 1. *mHealth solutions in form a web interface for providing better support to ASHAs* | |
| 2.1 | Average number of phone call by SEWA Rural’s ImTeCHO facilitator per ASHA per month towards providing support |
| 2.2 | Proportion of beneficiaries with high risk complications who received guidance from SEWA Rural’s helpline |
| 2.3 | Average number of phone call by SEWA Rural’s helpline per ASHA per month towards providing guidance for morbidity management |
| 2.4 | Proportion of ASHAs who were visited in field by ImTeCHO facilitator for providing support (mostly technology related) |
| 1. *mHealth solutions in form a web interface for providing motivating ASHAs* | |
| 3.1 | Average number of motivational announcements sent by SEWA Rural to ASHAs per month |
| 3.2 | Average amount of extra incentives paid by SEWA Rural per ASHA per month |
| 3.3 | Timeliness of ASHA incentive payment by PHC staff |
| 3.4 | Number of two-monthly mHealth facilitators review meetings held per year against expected |
| 3.5 | Number of quarterly district level review meetings held per year against expected |

Source of data: Work log of ImTeCHO facilitator for last three months of implementation period

1. WHO and UNICEF. Home visits for newborn child: a strategy to improve survival: WHO and UNICEF joint statement. 2009. [↑](#footnote-ref-2)
2. Government of Gujarat. Government resolution regarding roles of ASHA, Anganwadi worker and ANM. 24/9/2012. [↑](#footnote-ref-3)
3. # [**Victora CG**](http://www.ncbi.nlm.nih.gov/pubmed?term=Victora CG%5BAuthor%5D&cauthor=true&cauthor_uid=22999433), [**Barros AJ**](http://www.ncbi.nlm.nih.gov/pubmed?term=Barros AJ%5BAuthor%5D&cauthor=true&cauthor_uid=22999433), [**Axelson H**](http://www.ncbi.nlm.nih.gov/pubmed?term=Axelson H%5BAuthor%5D&cauthor=true&cauthor_uid=22999433), [**Bhutta ZA**](http://www.ncbi.nlm.nih.gov/pubmed?term=Bhutta ZA%5BAuthor%5D&cauthor=true&cauthor_uid=22999433), [**Chopra M**](http://www.ncbi.nlm.nih.gov/pubmed?term=Chopra M%5BAuthor%5D&cauthor=true&cauthor_uid=22999433), [**França GV**](http://www.ncbi.nlm.nih.gov/pubmed?term=França GV%5BAuthor%5D&cauthor=true&cauthor_uid=22999433), [**Kerber K**](http://www.ncbi.nlm.nih.gov/pubmed?term=Kerber K%5BAuthor%5D&cauthor=true&cauthor_uid=22999433), [**Kirkwood BR**](http://www.ncbi.nlm.nih.gov/pubmed?term=Kirkwood BR%5BAuthor%5D&cauthor=true&cauthor_uid=22999433), [**Newby H**](http://www.ncbi.nlm.nih.gov/pubmed?term=Newby H%5BAuthor%5D&cauthor=true&cauthor_uid=22999433), [**Ronsmans C**](http://www.ncbi.nlm.nih.gov/pubmed?term=Ronsmans C%5BAuthor%5D&cauthor=true&cauthor_uid=22999433), [**Boerma JT**](http://www.ncbi.nlm.nih.gov/pubmed?term=Boerma JT%5BAuthor%5D&cauthor=true&cauthor_uid=22999433). How **changes** in **coverageaffectequity** in **maternal** and **child healthinterventions** in **35Countdown** to **2015countries**: an **analysis** of **nationalsurveys**. [**Lancet.**](http://www.ncbi.nlm.nih.gov/pubmed/?term=How+changes+in+coverage+aff+ect+equity+in+maternal+and+child+health+interventions+in+35+Countdown+to+2015+countries%3A+an+analysis+of+national+surveys) 2012 Sep 29;380(9848):1149-56. doi: 10.1016/S0140-6736(12)61427-5.

   [↑](#footnote-ref-4)
4. Boerma JT, Bryce J, Kinfu Y, Axelson H, Victora CG. Mind the gap:equity and trends in coverage of maternal, newborn, and child healthservices in 54 Countdown countries. *Lancet* 2008; **371:** 1259–67. [↑](#footnote-ref-5)
5. Barros AJ, Ronsmans C, Axelson H, et al. Equity in maternal,newborn, and child health interventions in Countdown to 2015:a retrospective review of survey data from 54 ountries. *Lancet* 2012;**379:** 1225–33. [↑](#footnote-ref-6)
6. Countdown to 2012: *Building a Future for Women and Children: The 2012 Report*. 2012. [↑](#footnote-ref-7)
